# Intermittent Hypoxia Induces Greater Functional Breathing Motor Recovery as a Fixed Rather Than Varied Duration Treatment after Cervical Spinal Cord Injury in Rats

**DOI:** 10.1089/neur.2021.0004

**Published:** 2021-07-06

**Authors:** Aaron L. Silverstein, Warren J. Alilain

**Affiliations:** ^1^ Department of Neuroscience, University of Kentucky College of Medicine, Lexington, Kentucky, USA.; ^2^ Spinal Cord and Brain Injury Research Center, University of Kentucky College of Medicine, Lexington, Kentucky, USA.

**Keywords:** breathing motor plasticity, functional recovery, intermittent hypoxia, neuroplasticity, spinal cord injury

## Abstract

Intermittent hypoxia treatment (IH) has been shown to improve respiratory function in both pre-clinical animal models and human subjects following spinal cord injury (SCI), historically consisting of alternating and equal intervals of hypoxic and normoxic exposure. We describe such a procedure as fixed duration IH (FD-IH) and modulation of its severity, intermittency, and post-injury time-point of application differentially affects expression of breathing motor plasticity. As such, the established IH protocol exhibits similarity to instrumental conditioning and can be described as behavioral training through reinforcement. Findings from the field of operant conditioning, a form of more advanced learning, inspire the consideration that FD-IH protocols may be improved through exchanging fixed for varied durations of hypoxia between reinforcement. Thus, we hypothesized that varied duration intermittent hypoxia treatment (VD-IH) would induce greater breathing motor recovery ipsilateral to injury than FD-IH after cervical SCI in rats. To test this hypothesis, we treated animals with VD-IH or FD-IH for 5 days at 1 week and at 8 weeks following cervical SCI, then assessed breathing motor output by diaphragm electromyography (EMG) recording, and compared between groups. At 1 week post-injury, VD-IH-exposed animals trended slightly toward exhibiting greater levels of respiratory recovery in the hemidiaphragm ipsilateral to lesion than did FD-IH-treated animals, but at 8 weeks FD-IH produced significantly greater respiratory motor output than did VD-IH. Thus, these results identify a novel sensitivity of respiratory motor function to variations in the IH protocol that may lead to development of more effective treatments following SCI.

## Introduction

The majority of spinal cord injuries (SCIs) in the human population occur at the cervical level and can result in devastating respiratory motor dysfunction including diaphragm paresis and paralysis.^1^ Mechanical ventilation is often employed acutely to allow the patient to breathe despite the loss of endogenous inspiratory mechanisms, but it can result in atrophy of respiratory muscles and infection.^2^ Of those patients who improve enough to be eventually extubated, 20% may experience complications severe enough to necessitate re-intubation and a return to mechanical ventilation.^3^ Thus, strategies to improve respiratory function following cervical SCI are of immense clinical importance.

Intermittent hypoxia (IH) treatment has been experimentally applied in a rodent model of unilateral cervical SCI termed a C2 hemisection (C2Hx) to activate spared and typically latent pathways through episodically heightening the subjects' respiratory drive and increasing serotonergic signaling. This leads to a prolonged increase in their phrenic motor output known as long-term facilitation (LTF).^4,5^ Following experimental SCI, LTF can manifest in recovery of breathing motor function in the paralyzed hemidiaphragm, depending on various factors that include the duration, number, and post-injury time-point of IH treatment(s).^5–7^ However, at 12 weeks following C2Hx in rats, IH alone does not effectively induce breathing motor recovery.^8^ Even when IH is combined with treatment that degrades plasticity-inhibiting components of the extracellular matrix, breathing motor outcome is varied, and initially includes subjects with tonic diaphragmatic activity alongside those exhibiting coordinated inspiratory motor function.^8^

Additional work has also demonstrated that both sex and diversity of human ApoE genotype as expressed in mice affect receptiveness to LTF following IH.^9^ In 2013, IH was also studied in human subjects with incomplete SCI and was found to elicit increased minute ventilation (ventilatory LTF) for 30 min after acute exposure.^10^ However, these effects did not potentiate or become more prolonged following daily IH treatment. Collectively, although studies have clearly established the clinical relevancy of IH treatment, there is an identified need for optimization of IH protocols. Thus, our aim in this study was to further optimize existing IH paradigms to illicit greater, longer-lasting recovery.

It has been well established that modulation of the severity and intermittency of hypoxic exposure within fixed treatment differentially affects expression of LTF through multiple pathways.^11–13^ Further, LTF is sensitive to time-point of IH exposure.^14,15^ Because differences in hypoxia treatment severity and timing after injury profoundly impact respiratory motor outcome, it follows that variance of hypoxia duration could likewise be significant.

Most commonly employed IH protocols consist of 5-min exposures to hypoxia, interspersed with normoxic episodes of equal duration.^5,11^ Accordingly, we describe such a procedure as fixed duration intermittent hypoxia (FD-IH). FD-IH modifies breathing motor behavior leading to LTF, a neuroplastic process, and thus can be examined from the perspective of instrumental conditioning, wherein a behavioral response is trained through a subsequent outcome, or reinforcer. For a review of spinal cord plasticity and learning, please see work by Grau.^16^

If FD-IH is framed as an instrumental learning process, the targeted behavior of heightened respiratory motor output could be reinforced by either the acute improvement in the state of dyspnea sensed after respiratory compensation for hypoxia, or the ease of breathing experienced once the period of hypoxia ends and the subsequent bout of normoxia begins. In any case, the temporal proximity of response and outcome are key for learning to occur. For the purposes of this study, we have conceptualized the period of normoxia as the reinforcer of increased breathing motor activity stimulated by hypoxic exposure. In more advanced forms of learning, such as in operant conditioning, more consistent behavioral output can often be trained by application of variable time-intervals of reinforcement than by fixed intervals.^17^ Inspired by this concept, we have chosen to apply it to FD-IH treatment, predicting that a varied duration schedule of IH treatment could prove to be more adaptive for induction of functional breathing motor recovery following injury.

Therefore, using the fixed or varied pattern of the hypoxic duration as our independent variable, we hypothesized that varied duration intermittent hypoxia (VD-IH) treatment would induce a greater increase in respiratory motor output than FD-IH ipsilateral to injury following C2Hx in rats. However, any difference between the effectiveness of VD-IH and FD-IH in inducing LTF through utilization of the serotonergic innervation available at a 1-week time-point could be masked by the paucity of 5-hydroxytryptamine (5-HT) fibers acutely following hemisection—a paucity that is improved by 8 weeks post-injury.^14,15^ Thus, we tested our hypothesis at these two acute (1 week) and chronic (8 week) time-points to evaluate the comparative effects of VD-IH and FD-IH in both conditions of serotonergic availability.

## Methods

### Surgical procedure and C2 hemisection

All animal care and handling were carried out under strict compliance with the Institutional Animal Care and Use Committee guidelines at the University of Kentucky. Female, retired breeder, Sprague Dawley^®^ rats (Envigo-Harlan, Indianapolis, IN, USA) were anesthetized by intraperitoneal injection of a mixture containing ketamine (75 mg/kg body mass ([BM]) and xylazine (10 mg/kg BM). A C2Hx was performed according to previously described procedure.^8^ Briefly, the spinal cord was exposed at the second cervical level by incision through the overlying skin and muscular layers to allow for subsequent performance of laminectomy and then durotomy. Afterwards, a 27-gauge needle was inserted in the midline of the exposed spinal cord immediately caudal to the C2 spinal roots to span the extent of the cord's dorsoventral axis and then was moved laterally left to complete the hemisection.

Following closure of the surgical site, 0.6 mL of a 0.25 mg/mL saline solution of atipamezole was intramuscularly injected into the thighs bilaterally and repeated in 10 min if there was not arousal from anesthesia by that time. Once arousal was achieved, 1 mL of a 10μg/mL saline solution of buprenorphine and 1 mL of a 1.5 mg/mL saline solution of carprofen were injected subcutaneously (SQ), along with 3-6 mL of physiological saline. Further pharmacological aftercare consisted of another injection of buprenorphine at 12 h post-surgery and two carprofen injections SQ at 24 and 48 h post-surgery. All surgical animals recovered on a heated rack separate from naïve animals and were given *ad libitum* access to food and water and maintained on a standard light/dark cycle. Close monitoring of the surgical animals' well-being was maintained on a twice-daily basis until post-operative day 3, with supplemental saline administration, alternative diet provision, and other care according to the animals' needs. After 1 week, the animals were moved back to standard housing and their weight and well-being were monitored at least on a weekly basis.

### Animal groups and intermittent hypoxia training

**FIG. 1.**
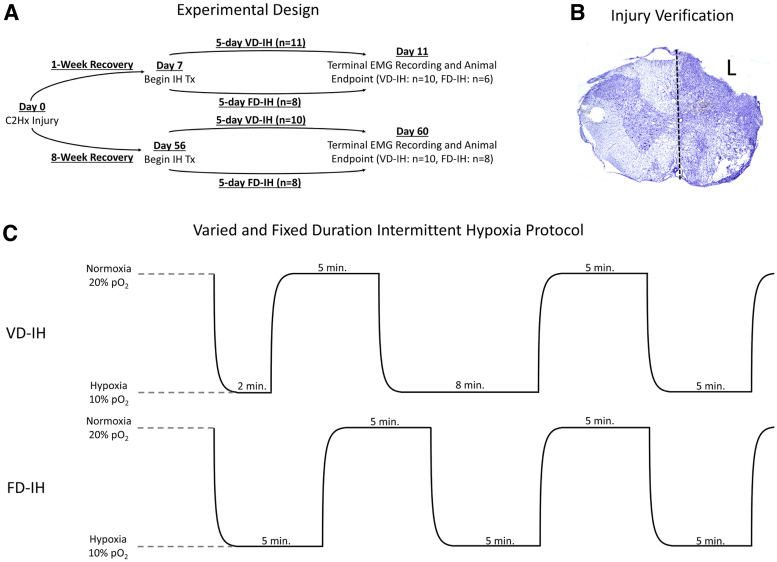
Experimental design, sample injury verification, and key methods. **(A)** Depiction of experimental design, time-points, treatment (Tx) groups, duration of IH treatment, and EMG recording end-point. **(B)** Example image of one of the complete C2Hx injuries verified by cresyl violet staining of an experimental rat's left lateral C2-level sectioned spinal cord. Animals displaying incomplete hemisections were taken out of the study. **(C)** Representative depiction of 3 cycles of VD-IH and FD-IH treatment. Each complete treatment day consisted of 12 cycles of normoxia/hypoxia. In FD-IH, periods of normoxia and hypoxia were equal to 5 min in duration, but in VD-IH, hypoxic periods varied from 2 to 8 min in integer values, with a cumulative average of 5 min/cycle each day of treatment. Of note, the durations of normoxia always lasted for 5 min across both treatments. The order in which the variable periods of hypoxia were administered in VD-IH was unique for each day, whereas the total time spent in normoxia and hypoxia was equal after each daily exposure and at the end of treatment. Transition from normoxia to hypoxia and from hypoxia to normoxia lasted an average of 160 sec/cycle across all treatments, exemplified by the sawtooth pattern seen above. C2Hx, C2 hemisection; EMG, electromyography; FD-IH, fixed duration intermittent hypoxia; IH, intermittent hypoxia; VD-IH, varied duration intermittent hypoxia.

After both 1-week and 8-week post-C2Hx time-points, animals were treated with FD-IH or VD-IH for 5 consecutive days, according to four unique experimental groups (Fig. 1A): 1-week VD-IH (*n* = 11); 1-week FD-IH (*n* = 8); 8-week VD-IH (*n* = 10); and 8-week FD-IH (*n* = 8). All treatment was conducted using the OxyCycler model A84XOV Atmosphere-controlled Chamber and accompanying software (Biospherix, Ltd., New York, USA). Animals were transported to an approved laboratory space to undergo treatment during the day, beginning between 0900 and 1500 h, and were returned to their vivarium once finished. Subjects were kept in their home cages without lids when placed into treatment chambers and were supplied with *ad libitum* food and water while inside.

For all treatment types, normoxia was defined as 20% and moderate hypoxia as 10% inspired O_2_ content. Each full hypoxia/normoxia cycle included both predetermined time at each set-point and an additional 160 sec for transition between hypoxia and normoxia. Each of the 12 cycles of FD-IH consisted of 5-min hypoxic periods and 5-min normoxic periods totaling 2 h and 32 min across all 12 cycles. The total duration of VD-IH was also equal to 2 h and 32 min, but the hypoxic periods' durations varied from 2 to 8 min in integer values in an order determined by a random number generator. Importantly, each day of VD-IH exposure entailed a new randomized order of the hypoxic periods' durations. For comparison, Figure 1C depicts three representative cycles of each treatment paradigm. In both FD-IH and VD-IH treatments, subjects spent a total of 60 min in hypoxia and 60 min in normoxia per daily 12-cycle treatment.

### Terminal electromyographic recordings

Immediately following final IH treatment day 5, animals were anesthetized with urethane solution in distilled water, injected intraperitoneally in two doses, 0.8 g/kg BM and 0.4 g/kg BM plus up to 0.06 g of additional urethane, respectively, separated by 1 h. Supplemental urethane was administered 30 min after the second injection only if pedal withdrawal and corneal blink reflexes indicated insufficient plane of anesthesia.

Once anesthetized, rats were placed inside a Faraday cage and a laparotomy was performed. A pair of 12 mm × 28 g Chalgren unipolar needle electrodes (CWE, Inc., Gilroy, CA, USA) was inserted into the dorsolateral quadrant of each costal hemidiaphragm. Before the beginning of baseline recording, the electrodes were re-inserted if initial placement lay outside of this region, or if the electrode became dislodged from the muscle. Additionally, an electrode was inserted into the abdominal musculature or the animal's hindfoot for each side's ground. A Power1401-3 Data Acquisition System and Spike2 8.08 software (CED, Cambridge, UK) along with a BMA-400 AC/DC Bioamplifier (CWE, Inc., Ardmore, PA, USA) were used to acquire and interpret the electromyography (EMG) signal.

After electrode insertion, the laparotomy site was covered with gauze pads and the Faraday cage doors were shut. A 30-min baseline recording was then completed, during which time no manipulation of the recording setup occurred. Afterwards, three nasal occlusions were performed on each animal and were noted along with the recording's time stamp. The animal's nostrils were occluded by hand to prevent nasal respiration for 15-sec durations, separated by sufficient time for the burst amplitude of the electromyographical recording to decrease to approximately pre-occlusion levels. After the final nasal occlusion, the recording was terminated and the animals were perfused with 1X phosphate-buffered saline (PBS), then 4% paraformaldehyde (PFA) in 1X PBS. Once perfusion was complete, each animal was dissected to remove its cervical spinal column.

### Data analysis

Data files were anonymized and arbitrary codes were assigned as labels so that the person quantifying was blinded to treatment group. Decoding occurred only after final quantification was completed for all recordings.

EMG data were analyzed using Spike2 8.08 software. For every EMG recording, measurements of left hemidiaphragm output amplitude ipsilateral to injury were taken from the integrated trace, which was generated from the direct current (DC)-removed, rectified, smoothed raw trace. The time constant for both the DC removal and smooth filter was 0.05 sec.

Amplitude of integrated diaphragm output was measured from the EMG recording at two time-points for left hemidiaphragms of each subject. At each time-point the measurement window was 6 sec in length. The first measurement window was centered on the time-point 15 min into the 30-min baseline and the second was positioned to include the highest amplitude burst generated during one of the three nasal occlusions, which occurred after the end of baseline. The average voltage amplitude of the diaphragm bursts for each time-point was measured.

Once these amplitudes were found, the baseline percentage of maximal output was calculated as described previously.^8^ The baseline amplitude measured was divided by the maximal amplitude for each hemidiaphragm and then multiplied by 100. For those animals that exhibited no hemidiaphragm activity on the left side ipsilateral to C2Hx at baseline, a value of 0 was yielded by this calculation, and for the one animal that exhibited no activity either at baseline or during nasal occlusion, an undefined value was returned. This undefined value was considered to be indicative of an absence of left hemidiaphragm activity and included in analysis as a 0 value.

### Verification of injury completeness

Following perfusion, spinal cord tissue (from 1-week VD-IH: *n* = 4; 1-week FD-IH: *n* = 3; 8-week VD-IH: *n* = 1; 8-week FD-IH: *n* = 0) was post-fixed in 4% PFA for 1–2 days and then cryoprotected. Then, the cervical spinal cord was embedded and cross-sectioned into 20-μm sections, using a cryostat, which were subsequently stored in a −20°C freezer.

For Nissl staining, slides were thawed on a slide warmer and placed into sequential solutions of 50% and 70% ethanol for 4 min each, then into 95%, and 100% ethanol solutions twice each for 3 min before placement into CitriSolv^®^ solution (Decon Labs, Inc., King of Prussia, PA, USA) for 5 min. Afterwards, slides were placed into the same ethanol solutions as before, but in reverse order.

Two quick rinses in distilled water were then completed before placement of the slides into a cresyl violet (Millipore Sigma, Darmstadt, DE, USA) solution of 0.1% in distilled water with glacial acetic acid for 3–5 min. Then, slides were placed into distilled water for up to 3 min and dipped into the same ethanol and CitriSolv^®^ solutions as previously described. After this, slides were mounted with Permount Mounting Medium™ (Electron Microscopy Sciences, Hatfield, PA, USA) and cover-slipped.

After staining was complete, slides were imaged using a BZ-X810 All-in-One Fluorescence Microscope under 40 × brightfield magnification (Keyence Corporation of America, Itasca, IL, USA). The most profoundly damaged section of tissue was identified for each animal analyzed and the level of the spinal cord was determined by the morphology of the dorsal and ventral horn gray matter. If visualized damage of gray and white matter reached completely from midline laterally to one side and from the dorsal to ventral aspects of the cross-section at a C2 or higher level, injury was considered complete (Fig. 1B). The EMG data gathered from subjects that appeared to have sustained incomplete injuries were excluded from final analysis.

### Statistical analysis

For all statistical comparisons, a *p*-value of 0.05 or less was accepted as denoting significance. This study compared differences in hemidiaphragmatic EMG output ipsilateral to injury following treatment with either VD-IH or FD-IH. Thus, a Student's *t* test was used for statistical analysis to compare differences between these two treatment groups at both 1 week and 8 weeks post-C2Hx. By design, there was no statistical comparison drawn between post-injury time-points or of any interaction between time-points and treatment types.

A Fisher's exact test of independence was secondarily utilized to compare categorical data consisting of the number of responders and non-responders between treatment groups at each time-point, utilized because of our 2 × 2 contingency table design and small sample sizes.

## Results

### FD-IH and VD-IH treatment at 1 week post-C2Hx does not induce differential respiratory motor activity

**FIG. 2.**
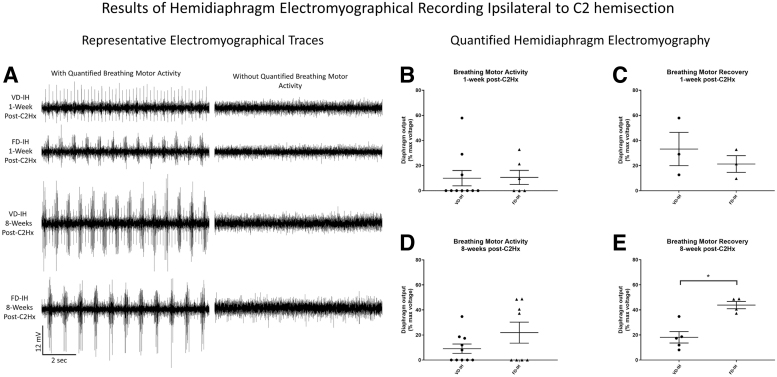
FD-IH induces greater breathing motor recovery than VD-IH at 8 weeks post-injury. **(A)** Raw EMG data from the diaphragm ipsilateral to injury from individual subjects designated as responders (left column) or non-responders (right column) during baseline recording following VD-IH or FD-IH at either 1 week or 8 weeks post-C2Hx, as labeled. **(B–E)** Data from EMG of the diaphragm ipsilateral to C2Hx, quantified as the percentage of the maximal amplitude of inspiratory bursting exhibited at baseline. (B) Quantification of diaphragm activity following VD-IH and FD-IH at 1 week post-C2Hx, including both responders and non-responders to treatment. No significant difference between treatments by Student's *t* test (*p* > 0.05). (C) Quantification of the inspiratory bursting activity of the responders to VD-IH and FD-IH at 1 week post-C2Hx. No significant difference between treatments by Student's *t* test (*p* > 0.05). (D) Quantification of diaphragm activity following VD-IH and FD-IH at 8 weeks post-C2Hx, including both responders and non-responders to treatment. No significant difference between treatments by Student's *t* test (*p* > 0.05). (E) Quantification of the inspiratory bursting activity of the responders to VD-IH and FD-IH at 8 weeks post-C2Hx. FD-IH treatment induced greater amplitude of diaphragm output than VD-IH treatment, by Student's *t* test (*p* = 0.001476). *Denotes significance, *p* < 0.01. C2Hx, C2 hemisection; EMG, electromyography; FD-IH, fixed duration intermittent hypoxia; IH, intermittent hypoxia; VD-IH, varied duration intermittent hypoxia.

Of those injured animals exposed to VD-IH treatment at 1 week post-C2Hx (*n* = 11), 1 was eliminated before analysis due to a dislodged electrode. Of the FD-IH-treated animals (*n* = 8), data from 2 subjects were eliminated from final statistical analysis, due to the animals' cresyl violet stain indicating incomplete hemisection. Of the remaining 10 VD-IH and 6 FD-IH subjects, 3 of the VD-IH animals (30%) exhibited quantifiable recovery of left hemidiaphragmatic activity, as compared with 3 out of 6 (50%) FD-IH animals (Fig. 2A). However, this difference in number of subjects exhibiting recovery was not significant by Fisher's exact test (*p* = 0.6066; Table 1).

**Table 1.** Comparison of the Number of Responders and Non-Responders after VD-IH and FD-IH

Time-point	Treatment	Number of subjects quantifiably recovered	Number of subjects not quantifiably recovered	Total subjects	Fisher's exact test of independence result
1 week	VD-IH	3	7	10	Not significant,
post-C2Hx	FD-IH	3	3	6	*p* = 0.6066
8 weeks	VD-IH	5	5	10	Not significant,
post-C2Hx	FD-IH	4	4	8	*p* = 1.0

C2Hx, C2 hemisection; FD-IH, fixed duration intermittent hypoxia; VD-IH, varied duration intermittent hypoxia.

Comparison of the left hemidiaphragmatic activity expressed by 1 week post-C2Hx animals (Fig. 2B) treated by VD-IH (mean [M] = 9.96%, standard error of the mean [SEM] = 6.11) and FD-IH (M = 10.62%, SEM = 5.61) yielded no significant difference by unpaired *t* test (t[14] = 0.0729, *p* = 0.9429). Subsequent analysis compared only the degree of recovery from subjects with non-zero hemidiaphragm output at baseline (Fig. 2C). We found no significant difference by unpaired *t* test (t[4] = 0.8069, *p* = 0.4650) between recovery amplitude induced by VD-IH (M = 33.18%, SEM = 13.23) and FD-IH (M = 21.23%, SEM = 6.67) at 1 week post-injury.

### FD-IH induces greater respiratory motor recovery than VD-IH at 8 weeks post-C2Hx

At 8 weeks post-C2Hx, 5 (50%) of the VD-IH-treated subjects (*n* = 10) and 4 (50%) of the FD-IH-treated subjects (*n* = 8) exhibited non-zero recovery (Fig. 2A). These results showed no difference by treatment type (Fisher's exact test: *p* = 1.0; Table 1).

Quantification of left hemidiaphragmatic activity (Fig. 2D) for VD-IH-treated animals (M = 9.06%, SEM = 3.52) and FD-IH-treated animals (M = 21.90%, SEM = 7.84) was compared by unpaired *t* test, and results trended toward significance (t[16] = −1.50469, *p* = 0.075944), indicating that FD-IH treatment induced greater hemidiaphragm activity than did VD-IH treatment. When data from subjects exhibiting non-zero recovery were analyzed (Fig. 2E), FD-IH (M = 43.80, SEM = 2.50) induced significantly greater amplitude recovery than did VD-IH (M = 18.13, SEM = 4.08) by unpaired *t* test (t[7] = −4.45556, *p* = 0.001476).

## Discussion

### Pattern sensitivity of IH treatment-induced LTF

The primary finding from this study suggests that FD-IH treatment of rats at a chronic, 8-week time-point post-C2Hx produces higher amplitude of breathing motor recovery than treatment with our VD-IH paradigm at the same time-point following injury. This was contrary to our initial hypothesis, clearly indicating that daily IH treatment following injury is not able to be improved by varying the duration of the hypoxic periods between 2 and 8 min. Nevertheless, our findings contribute to the field's understanding of the pattern-sensitivity of respiratory motor response to IH.

Our level of hypoxic exposure in both VD-IH and FD-IH was moderate in nature, consisting of 10% inspired oxygen, and likely leading to PaO_2_ of 40–54 mm Hg according to previously published findings,^13^ although arterial gas content was not a measured variable in our study. LTF induced by moderate IH is 5-HT_2_ receptor-dependent, termed the “Q” pathway, whereas more severe levels of hypoxic exposure (6–8% inspired O_2_) lead to adenosine A_2A_ and 5-HT_7_ receptor-dependent LTF through the “S” pathway.^6^ When the Q and S pathways are simultaneously activated, as in moderate sustained hypoxia (SH) for 25 min, LTF is not expressed.^13^

Succinctly, LTF is sensitive to the pattern of moderate IH applied, requiring intermittency of exposure to be induced.^12,13^ In our VD-IH treatment, our periods of hypoxia varied from 2 to 8 min in duration, and those with the longest durations of time might have masked the intermittency of the exposure, causing increased S pathway activation and subsequent crosstalk inhibition of the Q pathway. During the shortest duration of hypoxic periods, the heightened respiratory drive necessary for serotonergic release and subsequent Q pathway activation may have been only transiently activated, thus leading to no robust stimulation of the 5-HT_2_ receptors necessary for induction of LTF.^4^ As result, the combination of the 2–8 min hypoxic periods throughout our VD-IH treatment may have caused both ineffective Q pathway activation at one extreme and overactivation of the S pathway at the other, leading to less robust expression of LTF at the end of treatment than the 5-min periods of FD-IH. Future studies are needed to ascertain the upper and lower time limits of moderate hypoxic exposure that activate these competing pathways.

Because severe hypoxia treatment is known to induce sufficient S pathway activation to elicit robust LTF when applied to both SH and fixed duration IH paradigms, LTF appears to be less pattern-sensitive to severe levels of hypoxia than moderate levels.^11,13^ Thus, if severe hypoxia is applied to VD-IH treatment, it could produce greater LTF than moderate hypoxia applied in the same varied duration pattern. This provides another fruitful focus for future experimentation: the comparison of moderate and severe VD-IH treatments.

### Time-point sensitivity of IH treatment

Our intention in including an early, 1-week post-C2Hx time-point in this study was to identify whether our novel VD-IH might overcome barriers to functional recovery acutely after injury, but this proved not to be the case. One week after C2Hx, we observed no significant difference in breathing motor activity between FD-IH- and VD-IH-treated animals, although there was a slight, non-significant trend toward greater diaphragmatic activity at 1 week post-C2Hx in VD-IH-treated animals than in FD-IH-treated animals. It has been established that at 2 weeks post-C2Hx, there is low serotonergic input available in the phrenic motor nucleus and single exposure of rats to moderate acute IH treatment induces minimal LTF.^14^ However, daily IH treatment for 7 days at that same time-point induces robust, serotonin-independent LTF that might be mechanized by the adenosine A_2a_ receptor-mediated or S pathway.^15^ If VD-IH treatment pushes the S/Q balance toward the S pathway during longer hypoxic exposures and fails to efficiently induce Q pathway activation during the shorter periods of hypoxia, it would follow that VD-IH activates the S pathway more preferentially than the Q pathway. Thus, LTF following moderate VD-IH would likely become apparent at early post-injury time-points because the lower serotonergic innervation provides little substrate for the Q pathway. Perhaps our observed trend toward increased VD-IH-induced LTF at 1 week post-injury is a manifestation of this mechanism, although future experiments are necessary to measure adenosine and 5-HT receptor activation following VD-IH.

We chose our chronic, 8-week post-C2Hx time-point to allow for comparison of VD-IH and FD-IH in conditions wherein enhanced availability of serotonergic innervation would provide them with ample substrate for Q pathway activation.^14,15^ Indeed, we found that FD-IH induced significantly greater breathing motor recovery than did VD-IH at 8 weeks following C2Hx. This further supports an explanation of the comparatively lesser effectiveness of VD-IH as a result of crosstalk inhibition. If VD-IH does lead to greater S pathway stimulation than FD-IH, crosstalk inhibition of the serotonin-dependent Q pathway would render VD-IH-induced expression of LTF less sensitive to heightened 5-HT levels at the chronic time-point.

### Non-response to treatment

Heterogeneity of treatment response is present both in pre-clinical and clinical study populations and serves as a vital area of study.^18^ A better understanding of the factors causing differences in response even within syngeneic animal populations could identify novel treatment targets for translational and ultimately clinical application.^18^ The data from both 1-week and 8-week time-points in our study clearly depict heterogeneity of response within treatment groups, with some subjects exhibiting no hemidiaphragmatic activity ipsilateral to injury following treatment, whereas others showed significant recovery and breathing motor plasticity in the form of LTF. When statistical comparison of the number of responders versus non-responders was made between VD-IH and FD-IH treatment groups, no significant difference was determined. This would suggest that the non-response phenomenon is not sensitive to our IH pattern variation. Because genetic variation in laboratory rats is minimal and the degree and the type of cervical SCI in these animals was experimentally induced, it is also unlikely that response or non-response is due to any genetic predisposition for or against functional recovery or variance of the injury severity. Rather, it is likely to be caused by a factor or factors that are in common with the experimental procedure underwent by both VD-IH- and FD-IH-treated animals.

In this study, we chose to utilize diaphragm EMG recordings as our outcome measurement of breathing motor activity because of its more direct correlation to overall breathing function, which is important for clinical translation. The diaphragm EMG has been utilized in many studies of the breathing motor system, including many examining recovery of function following cervical SCI.^19–21^ However, the first studies^22^ to utilize IH to induce LTF measured breathing motor activity through a phrenic neurogram, a technique that may be more sensitive to smaller-scale changes in activation than a recording from the diaphragm innervated by the phrenic nerve. Subsequent studies exploring the signaling pathways of LTF and pharmacological stimuli for induction of similar plasticity also have utilized phrenic nerve recordings,^4,11,23,24^ perhaps again due to its greater sensitivity. For future studies on the pattern sensitivity of LTF, utilization of a phrenic neurogram as an outcome measure might detect lower levels of recovery than could an EMG, and might identify more of the true responders to IH treatment. This would allow a more robust comparison of the VD-IH- and FD-IH-induced levels of recovery.

### Considerations of instrumental conditioning

Our initial inspiration for studying VD-IH arose from our choice to construe IH treatment as a type of instrumental conditioning,^16^ considering the periods of normoxia as reinforcement of the increased breathing motor behavior exhibited during hypoxia. We then hypothesized that VD-IH would produce greater breathing motor recovery ipsilateral to hemisection than FD-IH because of findings from the field of operant conditioning indicating that more consistent behavioral output can be trained by use of variable rather than fixed time-intervals of reinforcement.^17^ However, we did not design our experiment to evaluate whether IH can be effectively treated as an instrumental conditioning procedure, and do not intend to interpret its results as such. Thus, our discussion has been primarily focused upon the physiological signaling pathways affected by VD-IH and FD-IH treatments and subject response to the end goal of improving breathing motor outcome following cervical SCI. Nevertheless, we wish to comment briefly on some additional perspectives on IH that might prove helpful for future work.

Instrumental conditioning relies upon the temporal contingency of the targeted behavior and the outcome of that behavior.^16^ Thus, IH treatment could potentially be improved by measuring the subject's minute ventilation using plethysmography during each interval of hypoxia and initiating the subsequent period of normoxia as the reinforcement in response to a predetermined frequency or amplitude of respiratory output. In our estimation, this would provide a valuable area of further research.

Further, IH could be considered as a series of spaced aversive hypoxic stimuli, separated by normoxia. This consideration leads us to suggest future experimentation evaluating varied intervals of normoxia within IH to ascertain whether such variance of the duration between hypoxic stimuli could affect respiratory motor response. We predict that this could give rise to a sort of learned helplessness, a phenomenon discussed by Seligman and Maier^25^ wherein subjects exposed to variable intervals of aversive stimuli eventually cease exhibiting escape behavior, even when there is opportunity. In rats exposed to periods of hypoxia spaced by varying durations of normoxia, this could lead to a decreased breathing motor “escape” response to hypoxia-induced dyspnea.

Additionally, work from Baumbauer and colleagues has shown that varied spaced sciatic nerve stimulation inhibits spinal cord learning in rats, whereas regularly spaced, or fixed, stimulation of the sciatic nerve or minor tailshock actually enhances learning, leads to *N*-Methyl-D-aspartic acid (NMDA) receptor activation, brain-derived neurotrophic factor (BDNF) release, protein synthesis, and even attenuates the spinal learning deficit induced by peripheral inflammation.^26–28^ Thus, we expect that future studies examining the possibility of variably spaced hypoxic stimulation will support the use of standard fixed intervals of normoxia within IH over varied normoxic intervals to induce more robust breathing motor output.

## Conclusion

This study introduces a novel type of pattern-sensitivity of LTF to moderate IH treatment in rats after cervical SCI. At chronic time-points following a C2Hx, 5-min-long fixed interval IH has here been shown to be more effective at inducing breathing motor recovery than our 2- to 8-min-long varied interval IH treatment. Because impairment of breathing motor function is an urgent therapeutic target in the human SCI population, development and optimization of treatment paradigms are crucial. IH is a promising therapeutic approach that is currently being utilized in clinical trials. This study and others that contribute to its understanding will allow it to be of increased benefit to future populations of people living with SCI.

## Supplementary Material

Supplemental data

## Abbreviations Used

5-HT: 5-hydroxytryptamine
BDNF: brain-derived neurotrophic factor
BM: body mass
C2Hx: C2 hemisection
EMG: electromyography
FD-IH: fixed duration intermittent hypoxia
IH: intermittent hypoxia
LTF: long-term facilitation
NMDA: *N*-Methyl-D-aspartic acid
OC: operant conditioning
PBS: phosphate-buffered saline
PFA: paraformaldehyde
SCI: spinal cord injury
SH: sustained hypoxia
SQ: subcutaneously
VD-IH: varied duration intermittent hypoxia
