# Supplementary material for: Intermittent Hypoxia Induces Greater Functional Breathing Motor Recovery as a Fixed Rather Than Varied Duration Treatment after Cervical Spinal Cord Injury in Rats
Source: Neurotrauma Rep. 2021 Jul 6;2(1):343–53. doi: 10.1089/neur.2021.0004 (PMC8310748; doi:10.1089/neur.2021.0004)
Supplement: Supplemental data [file Supp_Material.docx]

**Abbreviations Used**

IH: Intermittent hypoxia

SCI: Spinal cord injury

FD-IH: Fixed duration intermittent hypoxia

OC: Operant conditioning

VD-IH: Varied duration intermittent hypoxia

C2Hx: C2 hemisection

LTF: Long term facilitation

SH: Sustained hypoxia

BM: Body mass

SQ: Subcutaneous

PBS: Phosphate-buffered saline

PFA: Paraformaldehyde

EMG: Electromyographic
